# Heart rate variability is reduced in COVID‐19 survivors and associated with physical activity and fatigue

**DOI:** 10.14814/phy2.15912

**Published:** 2024-01-19

**Authors:** Michael H. Haischer, Lauren E. Opielinski, Lindsey M. Mirkes, Toni D. Uhrich, Rachel E. Bollaert, Michael Danduran, Marie Hoeger Bement, Linda B. Piacentine, Paula E. Papanek, Sandra K. Hunter

**Affiliations:** ^1^ Exercise Science Program Marquette University Milwaukee Wisconsin USA; ^2^ Athletic and Human Performance Research Center Marquette University Milwaukee Wisconsin USA; ^3^ Department of Physical Therapy Marquette University Milwaukee Wisconsin USA; ^4^ College of Nursing Marquette University Milwaukee Wisconsin USA; ^5^ Human Performance Assessment Core Marquette University Milwaukee Wisconsin USA; ^6^ Children’s Wisconsin Milwaukee Wisconsin USA

**Keywords:** autonomic dysfunction, heart rate variability, long COVID, physical activity, SARS‐CoV‐2

## Abstract

Reduced heart rate variability (HRV) and fatigue are common after COVID‐19 infection and both are potentially influenced by physical activity (PA). We compared resting HRV, PA from accelerometers and questionnaires, and self‐reported fatigue in 41 COVID‐19 survivors (~8 months postinfection, 38 ± 17 years) with 41 matched controls. Differences in HRV were observed on acceleration capacity (*p* = 0.041), deceleration capacity (*p* = 0.032), high‐frequency peak frequency (*p* = 0.019), absolute low‐frequency power (*p* = 0.042), relative very low‐frequency power (*p* = 0.012), SD2 (from Poincare plot; *p* = 0.047), and DFA2 (slope of long‐term detrended fluctuation analysis; *p* = 0.004). Fatigue was greater in COVID‐19 survivors (*p* < 0.001) with no differences in PA. Moderate‐vigorous physical activity (MVPA) (Standardized Beta = −0.427, *p* = 0.003) and steps per day (Standardized Beta = −0.402, *p* = 0.007) were associated with DFA2 in COVID‐19 survivors after controlling for age, sex, and body fat percentage. Fatigue was correlated to less MVPA (Spearman's rho = 0.342, *p* = 0.031) and fewer steps per day (rho = 0.329, *p* = 0.038) in COVID‐19 survivors, and was indirectly linked to HRV through these PA mediators (Estimate = −0.20; *p* = 0.040). We present a model showing the complex relations between HRV, PA, and fatigue that provides the foundation for strategies to improve outcomes and rehabilitation after COVID‐19 infection.

## 1 INTRODUCTION

The long‐term impact of SARS‐CoV‐2 infection (COVID‐19) on public health could be substantial because millions of people continue to experience lasting, debilitating effects. Originally termed “Long COVID” (*Nature*, 2020), the persistent health effects spanning multiple organ systems have been studied under a variety of names including post‐acute COVID‐19 syndrome (Nalbandian et al., 2021) and post‐acute sequelae of COVID‐19 (Al‐Aly et al., 2021), among others (A clinical case definition of post COVID‐19 condition by a Delphi consensus, 6 October 2021; Phillips & Williams, 2021; Thaweethai et al., 2023). More than 200 symptoms related to Long COVID are documented, with post‐exertional malaise, self‐reported fatigue, and heart palpitations among the most common (Thaweethai et al., 2023). Understanding the long‐term cardiovascular effects of COVID‐19 is important because of it's potential impact on morbidity and mortality. Survivors are at risk of heart attack, stroke, or heart failure due to the damage caused to the heart and blood vessels (Xie et al., 2022). Even when poor clinical outcomes are avoided, problems with dyspnea, fatigue, and exercise tolerance are often reported and limit exercise‐based rehabilitation (Alkodaymi et al., 2022; Aparisi et al., 2022). Dysfunction within the autonomic nervous system (ANS) is implicated in some of the cardiovascular complications from COVID‐19 due to its direct control over the heart (Dani et al., 2021; Raman et al., 2022). For this reason, noninvasively evaluating ANS function has become a common tool to evaluate patients both during and post‐COVID infection.

Heart rate variability (HRV) is a noninvasive biomarker that provides insight into ANS function and cardiovascular health (*Circulation*, 1996). A “healthy heart is not a metronome” with greater variability in the beat‐to‐beat rhythm associated with greater health (Shaffer & Ginsberg, 2017). The rhythm of the heart is modulated by the cardiac sinoatrial node through innervation of the sympathetic (SNS) and parasympathetic branches (PNS) of the ANS (Berntson et al., 1997). Thus, evaluation of HRV gives an indication of the variability in the beat‐to‐beat rhythm of the heart while providing insight into autonomic modulation of heart rate. Dysfunction within the ANS due to COVID‐19 could occur via direct viral invasion (Meinhardt et al., 2021) or autoimmunity and systemic inflammation (Raman et al., 2022). Regardless of the mechanism of action that leads to ANS damage, a host of studies have reported reduced HRV in people with COVID‐19 and shown associations with poorer health and worse clinical outcomes (Asarcikli et al., 2022; Barizien et al., 2021; Hasty et al., 2021; Kaliyaperumal et al., 2021; Mol et al., 2021; Pan et al., 2021; Solinski et al., 2022; Yin et al., 2023). One study of acute COVID‐19 patients related short‐term HRV (i.e., SDNN: standard deviation of normal RR intervals) to changes in C‐reactive protein, indicative of inflammation and disease state (Hasty et al., 2021). Thus, HRV seems to be able to noninvasively reflect the inflammatory state of COVID‐19 infection. Negative outcomes due to COVID‐19 in hospitalized patients have also been predicted by HRV (i.e., SDNN), with higher HRV associated with greater survival, and lower HRV predictive of admission to the intensive care unit (Mol et al., 2021). Collectively, reduced HRV is related to acute COVID‐19 severity (Hasty et al., 2021; Kaliyaperumal et al., 2021; Mol et al., 2021; Pan et al., 2021; Yin et al., 2023). Furthermore, ANS dysfunction does not appear to resolve in young (Solinski et al., 2022) and middle‐aged COVID‐19 survivors (Asarcikli et al., 2022), even in those experiencing mild lingering symptoms. Further study of Long COVID patients with and without fatigue suggests that the dysautonomia that may be observed through HRV could potentially explain that persistent symptom (Barizien et al., 2021). Strategies to improve ANS function and HRV in COVID‐19 survivors could help to minimize lingering health effects and maximize quality of life.

Importantly, physical activity (PA) behaviors may influence HRV (Henje Blom et al., 2009; Kluttig et al., 2010; Manser et al., 2021; Sandercock et al., 2005). Greater levels of PA are positively associated with increased HRV with studies in a variety of inactive and clinical populations showing a beneficial effect of regular exercise (Guiraud et al., 2013; Jurca et al., 2004; Ramirez‐Velez et al., 2020). However, reports of fatigue, which is common in the post‐acute phase of COVID‐19, is linked to reduced PA (Egerton et al., 2016; Rzepka et al., 2020). On the other hand, participation in PA and exercise may help reduce fatigue (Luctkar‐Flude et al., 2007; Puetz, 2006; Puetz et al., 2008). Furthermore, exercise may benefit the immune system (da Silveira et al., 2021) and shown that inactivity is associated with higher risk for severe clinical outcomes from COVID‐19 (Sallis et al., 2021). Taken together, clarifying the potential interrelationships of fatigue, PA, and HRV could have implications for both long‐term care of COVID‐19 survivors and exercise‐based rehabilitation. Even in apparently healthy survivors, small reductions in tolerance for physical exertion potentially related to compromised HRV could, in the long‐term, lead to reduced PA and further degradation of fitness and exercise tolerance. Helping patients within this expansive population avoid the downward spiral of physical and physiological function will go a long way towards minimizing the lasting burden on the health care system resulting from the COVID‐19 pandemic.

Thus, the primary aim of this study was to determine whether COVID‐19 survivors with a range of persistent symptoms had reduced HRV, lower PA, and greater fatigue, than matched controls. This cross‐sectional, case–control study was designed to test the *hypothesis* that HRV would be reduced, and PA would be lower, while self‐reported fatigue would be higher, in survivors when compared to healthy individuals matched on age, sex, height, and weight.

## 2 MATERIALS AND METHODS

The aims of the current study were accomplished by collecting and analyzing supine resting electrocardiograms (ECG) from which time‐domain, frequency‐domain, and nonlinear HRV variables were tested for between‐group differences for survivors and matched controls. Fatigue was reported via survey and PA data were obtained via survey and wearable accelerometry.

### 2.1 Participants

Data from 41 COVID‐19 survivors (COV) and 41 age‐, sex‐, height‐, and weight‐matched control participants (CON) were collected as part of a larger, single‐site study of COVID‐19 survivors between November 2020 and November 2022. Each group within the convenience sample consisted of 12 males (age range: 20–66 years) and 29 females (age range: 19–69 years). Survivors were included in the study if they had self‐reported a positive diagnosis of COVID‐19, (via a positive test when available and/or medical diagnosis) and were at least 2 months past the date of the positive test or medical diagnosis. Control participants were recruited at the same time during the pandemic. To be included as CON, participants reported never experiencing any symptoms related to COVID‐19 and had never tested positive for COVID‐19. Pregnant women were excluded from the study, as were volunteers that were determined to be taking medication that could potentially impact HRV (e.g., beta‐blockers). Individuals with conditions or diseases that would hinder their ability to perform various tests and measures involved in the larger study (e.g., myocardial infarction in the last 12 months, pulmonary embolism, musculoskeletal issues, fibromyalgia, active cancer) were also excluded. Participation in the study involved 1 day of in‐person data collection followed by a week of monitoring of PA via a wearable device. All procedures were approved by the Marquette University Institutional Review Board and all participants provided written informed consent.

Each participant attended a single session of testing in the laboratory. Participants were required to refrain from exercise, caffeine, and alcohol for 12 h prior to data collection and all were screened for COVID‐19 symptoms and confirmed to test negative via nasopharyngeal swab or saliva test on the day of the appointment to be included in the study. Blood pressure was collected via automatic digital blood pressure monitor (Omron Healthcare HEM‐907XL, Kyoto, Japan) with participants seated at rest in a quiet room. Height was recorded via stadiometer (Seca, Hamburg, Germany). Weight and body‐fat percentage were obtained by multifrequency quadripolar bioelectrical impedance analysis (Tanita MC780‐U, Arlington Heights, IL, USA).

### 2.2 COVID‐19 symptoms

COVID‐19 survivors were surveyed about their symptoms during initial infection from SARS‐CoV‐2 and at the time of testing using a study‐developed tool. This tool included a 22‐item checklist of the most common COVID‐19 symptoms identified by the Centers for Disease Control and Prevention at the time, along with an additional open‐ended “Other symptoms” item (Table S1). Symptom severity during both timepoints was scored from 0 to 4 [0 = None; 1 = Low (1–3 symptoms); 2 = Moderate (4–7); 3 = High (8–11); 4 = Very High (12–17)] to facilitate analyses of both total number of symptoms and ranked severity.

### 2.3 HRV

Five‐minute, 12‐lead supine resting ECG were recorded from all 82 participants (100 Hz; GE Cardiosoft v6.73, Boston, MA, USA) in a darkened, quiet room. Recordings began after the participants were resting supine for at least 10 minutes and when the signal was observed to be stable. Non paced (free) breathing was allowed to maximize comfort. Data was exported to Kubios Premium HRV software (version 3.5.0, Kuopio, Finland) for processing and analysis (Tarvainen et al., 2014). Ectopic beats were identified and corrected using the beat classification algorithm within the software that replaces the corrupted RR interval times with interpolated values (Lipponen & Tarvainen, 2019). The complete list of HRV variables of interest includes those from time‐domain, frequency‐domain, and nonlinear analyses (Table 1). The HRV spectrum was estimated in the software using fast Fourier transform‐based Welch's periodogram method (very low frequency = 0 to 0.04 Hz; low frequency = 0.04–0.15 Hz; high frequency = 0.15 to 0.40 Hz). For details on these methods and metrics, refer to the software website (https://www.kubios.com/hrv‐analysis‐methods/) (Tarvainen et al., 2014).

**TABLE 1:** Output variables from heart rate variability analysis.

Analysis domain	Variables of interest
Time	Mean RR interval, SDNN (standard deviation of RR interval), mean heart rate, RMSSD (root mean square of successive differences), NN50 (number of RR interval pairs that differ more than 50 ms), pNN50 (NN50 divided by total RR intervals), triangular index, TINN (width of RR interval histogram), AC (acceleration capacity), and DC (deceleration capacity)
Frequency	Peak frequencies, absolute powers, relative powers, and normalized powers of frequency bands (very low frequency: VLF, low frequency: LF, high frequency: HF), total spectral power, and LF/HF power ratio
Nonlinear	SD1, SD2 (in Poincare plots, standard deviations perpendicular and along line‐of‐identity, respectively), SD2/SD1, approximate entropy, sample entropy, correlation dimension, DFA1, and DFA2 (slope of short‐term and long‐term detrended fluctuations, respectively)

### 2.4 Fatigue and PA

Self‐reported fatigue was assessed using the Functional Assessment of Chronic Illness Therapy—Fatigue Scale (FACIT), with lower scores corresponding to greater fatigue (Yellen et al., 1997). PA levels were determined from questionnaire and also from accelerometry over 7 days. Current PA (COV and CON) and PA prior to infection (COV only) was estimated retrospectively from the International PA Questionnaire (IPAQ) (Craig et al., 2003). Wearable devices (ActiGraph GTX3, Pensacola, FL, USA) were distributed to the participants to measure accelerations as a proxy for PA on seven consecutive days following in‐person data collection. Participants were instructed to wear the devices on the provided belt over the nondominant hip upon waking until going to sleep. After the accelerometer was returned to the laboratory, data was downloaded, processed, and analyzed in ActiLife software (version 6.13.4) for sedentary time, light and moderate‐vigorous PA time, and step count. A minimum wear time of 600 min per day was required for inclusion of accelerometry data.

### 2.5 Statistical analysis

Data were reported as means and standard deviations. To verify control matching, independent samples *t*‐tests were performed between the two groups (COV and CON) on age, height, weight, body fat percentage, systolic blood pressure, diastolic blood pressure, and resting heart rate. Normality of the HRV, PA, and fatigue data was assessed via visual inspection of Q‐Q plots and evaluation of skewness and kurtosis. Due to abnormal data distributions, group comparisons between COV and CON were completed using Mann–Whitney *U* tests. Adjustments to the threshold for significance were not made for multiple comparisons due to the exploratory nature of the study. Effect sizes for comparisons were reported as Hedges' g (Hedges & Olkin, 1985) calculated using the recommendations and spreadsheet provided by Lakens (Lakens, 2013). Common language effect sizes (CL) (McGraw & Wong, 1992) were also calculated using the same tool to facilitate practical interpretation of the probability of superiority (Grissom & Kim, 2005). Briefly, CL expresses the probability that a randomly chosen individual from one group (e.g., COVID survivors) will have a higher value than an individual from the other group (e.g., controls). Where significant differences in HRV were found, regression analyses were performed on COV only to evaluate the impact of fatigue, PA, total symptoms, and ranked symptom severity on HRV with consideration given to known covariates (i.e., age (Thayer et al., 2010), sex (Koenig & Thayer, 2016), body fat percentage (Chen et al., 2019; Sjoberg et al., 1985)). Spearman's rho correlations with 1000 replicate sample bootstrapping were used as an additional analysis to describe the relationship between fatigue and any PA variables that were significant predictors of HRV. Finally, mediation analyses were performed to investigate how the PA variables cumulatively impact the influence of fatigue on HRV. Mediation analyses were performed in JASP 0.17.3 (JASP Team, Amsterdam, Netherlands), while all others were done in SPSS version 26 (IBM, Armonk, NY, USA) with the alpha level set at 0.05.

## 3 RESULTS

As expected, age, sex, height, and weight did not differ between COV and CON (Table 2). Additionally, no difference was found between groups in body fat percentage, resting blood pressure, or resting heart rate (Table 2).

**TABLE 2:** Matched anthropometric and resting cardiovascular measures between covid‐19 survivors and controls.

	COV (*n* = 41)	CON (*n* = 41)	Sig. (*p* Value)
Sex	F, 29; M, 12	F, 29; M, 12	–
Months since illness	7.71 ± 3.53	–	–
Age (y)	38 ± 17	38 ± 17	0.905
Height (cm)	169.7 ± 6.7	169.6 ± 7.0	0.952
Weight (kg)	70.1 ± 13.2	68.1 ± 13.7	0.507
Body fat (%)	25.7 ± 8.9	24.6 ± 8.8	0.597
Systolic BP (mmHg)	121 ± 16	123 ± 17	0.710
Diastolic BP (mmHg)	73 ± 11	74 ± 10	0.687
Resting heart rate (bpm)^a^	64 ± 14	62 ± 9	0.349

Abbreviation: bpm, beats per minute.

^a^ From electrocardiogram.

**FIGURE 1:**
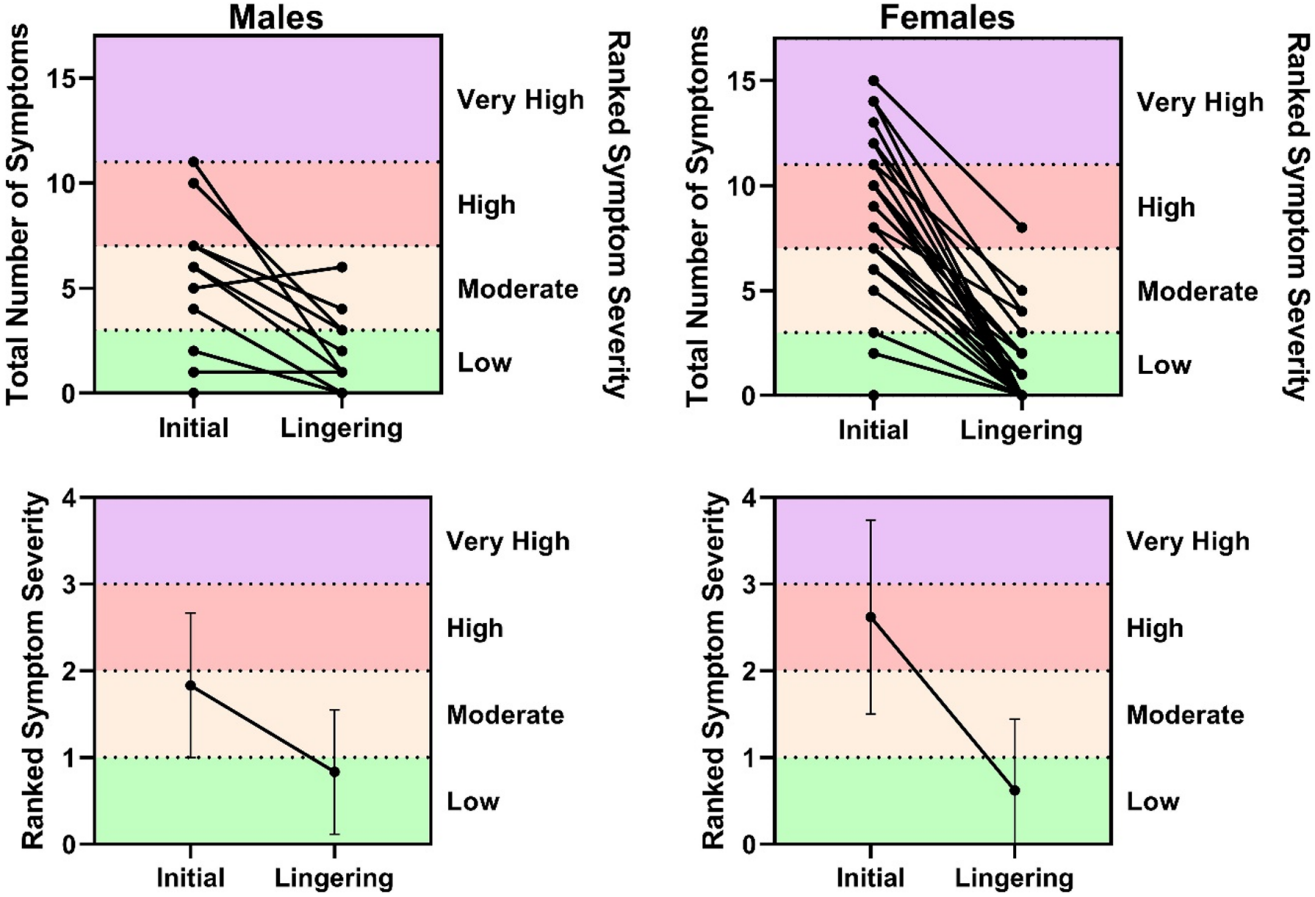
Symptomology of COVID‐19 survivors. Only one male participant reported more lingering symptoms than number of symptoms at initial infection.

Survivors reported a wide range of experiences with COVID‐19 both in total number of symptoms and ranked symptom severity (Figure 1). None of the survivors required hospitalization for their acute illness. 51% (*N* = 21) of the cohort reported lingering symptoms at the time of testing, almost 8 months after initial infection on average. The most common symptoms during initial COVID‐19 infection for males were fatigue (67%, *N* = 8), muscle pain (58%, *N* = 7), headache (42%, *N* = 5), loss of taste (42%, *N* = 5), and runny nose (42%, *N* = 5). Females commonly experienced headache (86%, *N* = 25) and fatigue (83%, *N* = 24), as well as loss of smell (69%, N = 20) during initial infection. The most common lingering symptoms in both males and females were fatigue (M: 25%, *N* = 3; F: 21%; *N* = 6), brain fog (M: 25%, *N* = 3; F: 17%, *N* = 5) and headache (M: 17%, *N* = 2; F: 14%, *N* = 4). Additionally, men noted a persistent loss of taste (17%, *N* = 2).

### 3.1 Matched control analyses

Of the 30 HRV variables compared between COV and CON, significant differences were found across all three domains of analysis (Table 3, Table S2).

**TABLE 3:** Differences in HRV between COVID‐19 survivors and controls.

Analysis domain	Variable^a^	COV (*n* = 41)	CON (*n* = 41)	Sig. (P)	Hedges' g	CL (%)
Time	AC (ms)	−19.6 ± 13.8	−26.4 ± 16.4	0.041	0.45	62.4
	DC (ms)	20.7 ± 17.1	30.8 ± 24.0	0.032	0.48	63.4
Frequency	HF peak frequency (Hz)	0.233 ± 0.059	0.202 ± 0.049	0.019	0.57	65.7
	Absolute LF power (ms^2^)	1071 ± 1471	2462 ± 3517	0.042	0.51	64.2
	Relative VLF power (%)	6.65 ± 5.78	3.86 ± 3.51	0.012	0.58	66.0
Nonlinear	SD2 (ms)	52.0 ± 28.8	70.9 ± 44.9	0.047	0.50	63.8
	DFA2	0.297 ± 0.144	0.214 ± 0.090	0.004	0.68	68.8

^a^ For variable descriptions, see Table 1.

Abbreviations: CL, common language effect size; Hz, hertz; ms, milliseconds.

**FIGURE 2:**
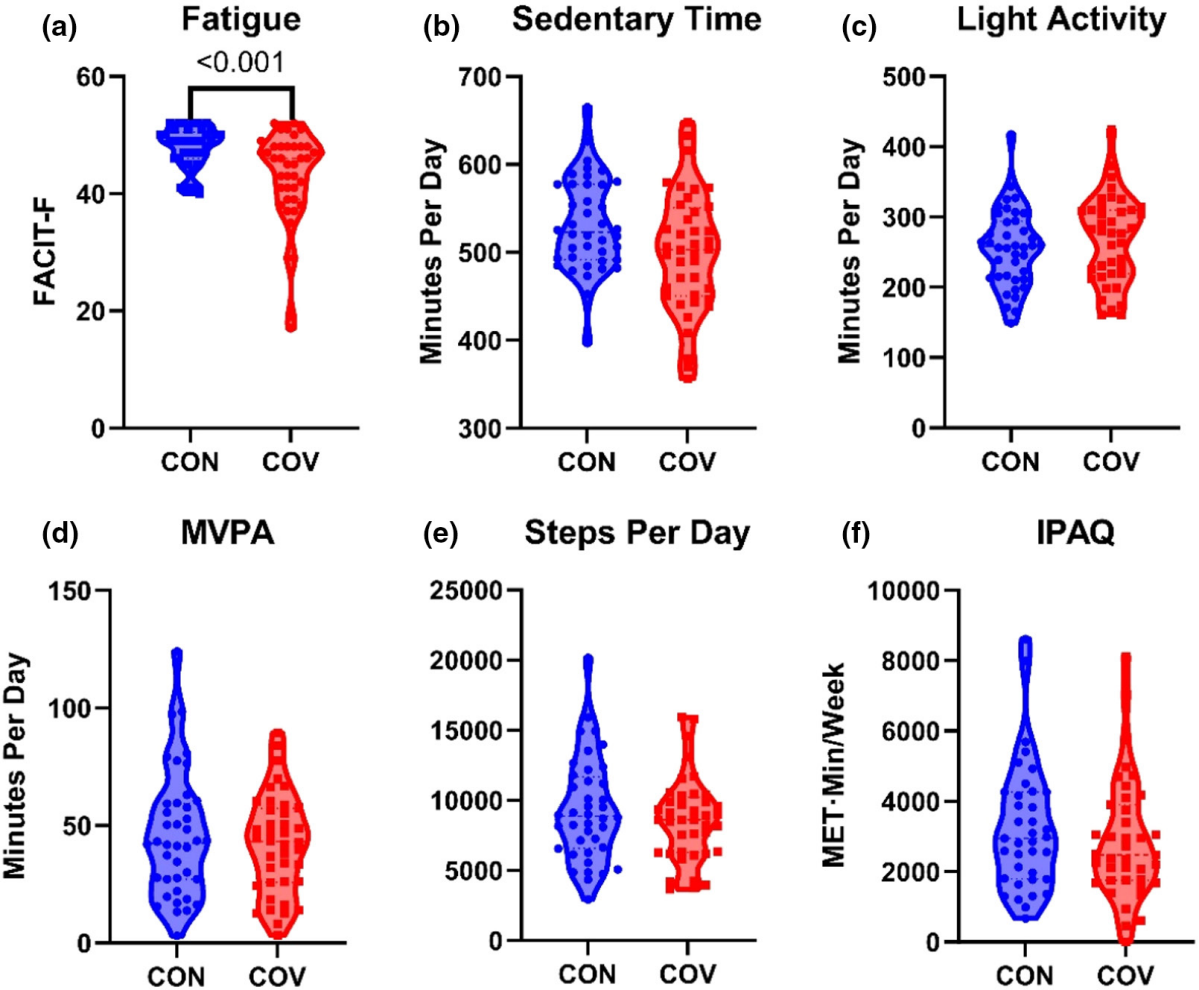
Self‐reported fatigue and physical activity comparisons. (a) Self‐reported fatigue from the functional assessment of chronic illness therapy—fatigue scale (0–52 range; lower scores indicate greater fatigue) (Yellen et al., 1997). (b–e) Activity variables collected from the ActiGraph wearable device. MVPA: moderate/vigorous physical activity. (f) Current self‐reported physical activity from the International Physical Activity Questionnaire (Craig et al., 2003).

From the FACIT, greater levels of fatigue were reported by COV (42.9 ± 8.0) than CON (47.9 ± 3.5; *p* < 0.001, *g* = 0.80, CL = 71.7%) (Figure 2a). Note that lower scores indicate greater fatigue.

Average accelerometer wear time did not differ between groups (COV: 811 ± 71 min/day; CON: 834 ± 73 min/day). All participants wore the accelerometer for at least five of the seven prescribed days. Sedentary time differed with COV (501 ± 68 min/day) spending about 1 h less per day on average than CON (557 ± 171 min/day; *p* = 0.023, g = 0.43, CL = 62.0%). However, this was driven by one female CON participant heavily skewing the data and analysis without the outlier showed no between‐group differences in sedentary time. Thus, no significant differences were found between groups in the PA variables (Figure 2b–f). Data for male and female subgroups are presented in Table 4.

**TABLE 4:** Fatigue and physical activity in male and female covid‐19 survivors and controls.

	Male COV (*n* = 12)	Male CON (*n* = 12)	Female COV (*n* = 29)	Female CON (*n* = 29)
FACIT^a^	43.3 ± 9.5	47.7 ± 2.5	42.7 ± 7.5	48.0 ± 3.8
Sedentary time (min/day)^b^	491 ± 76	551 ± 51	505 ± 66	559 ± 202
Light activity (min/day)^b^	251 ± 62	258 ± 54	276 ± 62	255 ± 56
MVPA (min/day)^b^	45 ± 25	51 ± 34	41 ± 20	44 ± 23
Steps per day^b^	9052 ± 3972	9999 ± 4833	8459 ± 2557	9023 ± 3072
Current IPAQ (MET·min/week)	3215 ± 1943	2270 ± 1288	2724 ± 1610	3703 ± 1837
Pre‐infection IPAQ (MET·min/week)	8046 ± 4808	–	3322 ± 2015	–

Abbreviations: FACIT, functional assessment of chronic illness therapy—fatigue scale; IPAQ, international physical activity questionnaire; MVPA, moderate/vigorous physical activity.

^a^ Possible Range: 0–52; lower scores indicate greater fatigue.

^b^ From wearable device.

### 3.2 Regression analyses of HRV in COV

Significant differences (*p* < 0.05) were found between COV and CON in AC, DC, HF band peak frequency, absolute LF power, relative VLF power, SD2, and DFA2, thus these variables were used as dependents in subsequent multivariate regression analyses. Fatigue, PA variables, number of symptoms, and ranked symptom severity were each entered in their own model as independent variables with covariates including age, sex, and body fat percentage. Controlling for covariates, average moderate/vigorous physical activity (MVPA) per day (42 ± 21 min, standardized Beta = −0.427, *p* = 0.003, 95% Confidence Interval = −0.005 to −0.001, model *R* ^2^ = 0.454) and steps per day (8637 ± 3008 steps, standardized Beta = −0.402, *p* = 0.007, 95% CI = −3.3·10^−5^ to −0.6·10^−5^, model *R* ^2^ = 0.431) collected from the wearable accelerometers were both significantly predictive of DFA2. No other fatigue, PA, or symptomology variables were associated with HRV metrics in this cohort of COVID‐19 survivors that were observed to be different than CON (Table S3). Correlation analysis revealed greater self‐reported fatigue in COV was significantly related to less MVPA (Spearman's rho = 0.342, *p* = 0.031, 95% CI = 0.025–0.597) and fewer steps per day (rho = 0.329, *p* = 0.038, 95% CI = 0.010–0.588).

Subsequent COV mediation analysis showed a significant total indirect effect of the PA variables in mediating the influence of fatigue on HRV (Estimate = −0.20; *p* = 0.040; 95% CI = −0.483 to −0.034). This effect was also present when using data from the entire sample (both COV and CON) (Estimate = −0.088; *p* = 0.050; 95% CI = −0.215 to −0.019). Thus, average MVPA and steps per day cumulatively mediate the relationship between fatigue and HRV.

## 4 DISCUSSION

This study is the first to our knowledge that demonstrates associations between PA and HRV variables in COVID‐19 survivors. Importantly, only HRV variables were different between COVID‐19 survivors and matched controls after controlling for age, sex, and body fat percentage within the regression models. Further novelty lies in that we correlated self‐reported fatigue to PA variables that were associated with HRV and performed follow‐up mediation analysis, the results of which showed that PA influences the relationship between fatigue and HRV. We also confirmed that HRV was reduced in people who had previously had a diagnosis of COVID‐19 compared with those without diagnosis, even 8 months after initial infection.

Our results demonstrated differences between groups in the time‐, frequency‐, and nonlinear HRV analysis domains indicating reduced HRV in COV compared to CON. Heart rate acceleration (AC) and deceleration capacity (DC) of survivors were less than CON. These findings suggest that COVID‐19 survivors may be less capable of increasing or decreasing their heart rate to meet physiological demands or recover from exertion. This result is disconcerting in light of the previous findings on DC showing that in a large cohort of individuals after myocardial infarction, reduced DC was the most powerful predictor of mortality (Bauer et al., 2006). Decreased DC has also been associated with increased risk of arrythmias post‐myocardial infarction (Liu et al., 2020). Observed differences in frequency‐domain powers could also signal a shift in modulation between the branches of the ANS. Aligning with other case–control studies of COVID‐19 survivors, our data potentially points to parasympathetic dominance of the ANS (Asarcikli et al., 2022; Solinski et al., 2022). Interestingly, Asarcikli and colleagues observed increased HRV in their COVID‐19 survivor cohort 12–26 weeks after initial COVID‐19 infection when compared to controls (Asarcikli et al., 2022). Further studies suggested that this could be the result of a compensatory anti‐inflammatory response through the vagal‐cholinergic pathway of the parasympathetic branch (Pavlov & Tracey, 2017). Thus, while reduced HRV may be more typically associated with COVID‐19 infection, increased HRV may also be observed depending on the current inflammatory state of the individual and modulation by the PNS. In a later investigation of critically ill COVID‐19 patients, it was proposed that this shift towards parasympathetic dominance could also be potentially related to weakening of the sympathetic nervous system (Aragon‐Benedi et al., 2021).

At first, our results and others appear to conflict with the results of a study by Stute et al., which showed reduced autonomic function characterized by increased sympathetic activity (Stute et al., 2021). However, conclusions in that study were primarily based on measurements from muscle sympathetic nerve activity and HRV analysis results also showed increased parasympathetic modulation. Indeed, a recent study suggests that while dysfunctions of both ANS branches may be observed, parasympathetic impairment seems to appear more often and could potentially account for disability post‐COVID‐19 infection (Zanin et al., 2023). Lastly, decreased SD2 and increased DFA2 from the nonlinear analyses in our study indicate a more ordered (i.e., less random) pattern in beat‐to‐beat intervals (Shaffer & Ginsberg, 2017). This observed reduction in the complexity within the time‐series was also noted in a previous case–control study, though with different nonlinear metrics (Solinski et al., 2022), and may be interpreted as another finding potentially indicative of ANS dysfunction in COVID‐19 survivors. Even where significant between‐group differences in our study were not observed, it is interesting to consider the COVID survivors' HRV data in relation to normative values and to the CON cohort. For example, SDNN in COV was lower than 50 ms, which has been used as a cutoff to stratify cardiac risk and classify patients as “unhealthy” from 24‐h recordings, while CON was above this threshold (Table S2) (Kleiger et al., 1987). While RMSSD was also not significant between COV and CON, the mean difference of 11 ms could be clinically meaningful and indicate reduced vagal mediation of HRV (Shaffer et al., 2014). Further, the total power observed in COV survivors was more than 40% less than that observed in CON, on average. Reduced total power has been linked to autonomic neuropathy in individuals with diabetes (Malik, 1996) and is indicative of reduced autonomic activity overall.

Further analyses of the survivor cohort revealed that MVPA and steps per day obtained from wearable accelerometry were independent, predictors of HRV (i.e., DFA2), when controlling for age, sex, and body fat percentage. Thus, higher activity levels may benefit COVID‐19 survivors by encouraging a more complex beat‐to‐beat rhythm. Unlike previous literature that related physical inactivity to severe COVID‐19 outcomes (Sallis et al., 2021), we found that pre‐COVID PA (via IPAQ) was not a predictor of, rather a modifier of HRV. Regardless, existing literature across all ages shows a beneficial effect of PA or exercise on HRV (Guiraud et al., 2013; Henje Blom et al., 2009; Jurca et al., 2004; Kluttig et al., 2010; Ramirez‐Velez et al., 2020; Sandercock et al., 2005). Importantly, self‐reported fatigue was inversely correlated to both predictors of HRV (i.e., MVPA and steps per day). Furthermore, MVPA and steps per day were shown to collectively mediate the influence of fatigue on DFA2. With greater fatigue relating to less PA, less activity being associated with ANS dysfunction as observed through HRV, and activity mediating the relationship between fatigue and HRV, the interplay of these variables could lead to a continued downward spiral of function in COVID‐19 survivors. Because exercise and PA are key lifestyle habits that generally increase HRV over time (Guiraud et al., 2013; Henje Blom et al., 2009; Jurca et al., 2004; Kluttig et al., 2010; Ramirez‐Velez et al., 2020; Sandercock et al., 2005), reflecting increased fitness, there are implications of this investigation related to both individual exercise testing and prescription and broader public health strategies and interventions.

### 4.1 Clinical and exercise implications

First, reduced HRV could result in responses to exercise that are different than what is typically observed in a healthy population. For example, reduced acceleration and DC of heart rate may extend the amount of time it takes to adapt to greater workloads at the onset of exercise and prolong recovery to physiological baseline afterwards. Thus, selecting exercise testing protocols that involve lower intensities and more gradual ramps in workload is advisable as it will be safer and could improve data collection by allowing participants to move further through graded exercise testing stages. These approaches are also relevant to self‐reported fatigue because adherence to an exercise program is likely to be maximized if overexertion (whether real or perceived) through excessive intensity or volume can be avoided (Collado‐Mateo et al., 2021).

From a public health perspective, the findings of our study suggest that policies and interventions designed to address both fatigue and increasing PA in COVID‐19 survivors may be more effective at improving health than strategies that focus on one of those targets in isolation. One strategy could be to increase public awareness messaging of reduced fatigue as one of the possible benefits of regular exercise and PA, which has been observed in other clinical populations such as cancer (Hilfiker et al., 2018; Wagoner et al., 2021) and chronic fatigue syndrome (Galeoto et al., 2018). Overall, our data suggests that in COVID‐19 survivors, ANS function, fatigue, and PA levels may benefit from gradual, progressive exercise rehabilitation programs.

### 4.2 Study limitations

The cross‐sectional design of this study is limitied in that we cannot determine within‐survivor changes in HRV that occurred specifically due to COVID‐19. However, the rigorous nature of our control matching, similarities in resting cardiovascular measures (i.e., blood pressure and heart rate) between groups, and presence of between‐group differences across multiple HRV metrics provided evidence of long‐term impairment in ANS function in COVID‐19 survivors even 8 months post infection. Another potential limitation of this study is the short, five‐minute ECG recording that was used for HRV analysis because longer‐term recordings that would provide insight into ANS function during wake and sleep were not feasible. Paced breathing was also not adopted during ECG measurements to maximize comfortability and relaxation of participants during the short‐term recordings, and thus our findings should be interpreted with the understanding that we did not control for respiratory sinus arrhythmia. Thirdly, our description of symptoms and ranked severity was relatively simple and doesn't account for specific symptoms. While symptom numbers and ranked severities did not predict HRV, there may be certain acute COVID‐19 symptoms that are more likely to precede post‐acute ANS dysfunction. As a related point, no hospitalizations were required for acute COVID‐19 within our sample of survivors so these findings may not generalize to the subpopulation of survivors that were extremely ill and required in‐patient care. Next, providing an accelerometer to measure activity time may have promoted a change in activity behaviors. However, participants were told to go about their daily activities as normal. Similarly, retrospectively assessing PA through the IPAQ is potentially subject to recall bias but self‐reporting was the only possible approach due to the nature of the study. Finally, the current study was conducted on volunteers that almost all identified as Caucasian (80 of 82 participants). As differences in PA habits have been reported between races and ethnicities (Saffer et al., 2013), it is reasonable to expect that these findings may not translate across different racial and ethnic groups.

### 4.3 A theoretical model and conclusions

**FIGURE 3:**
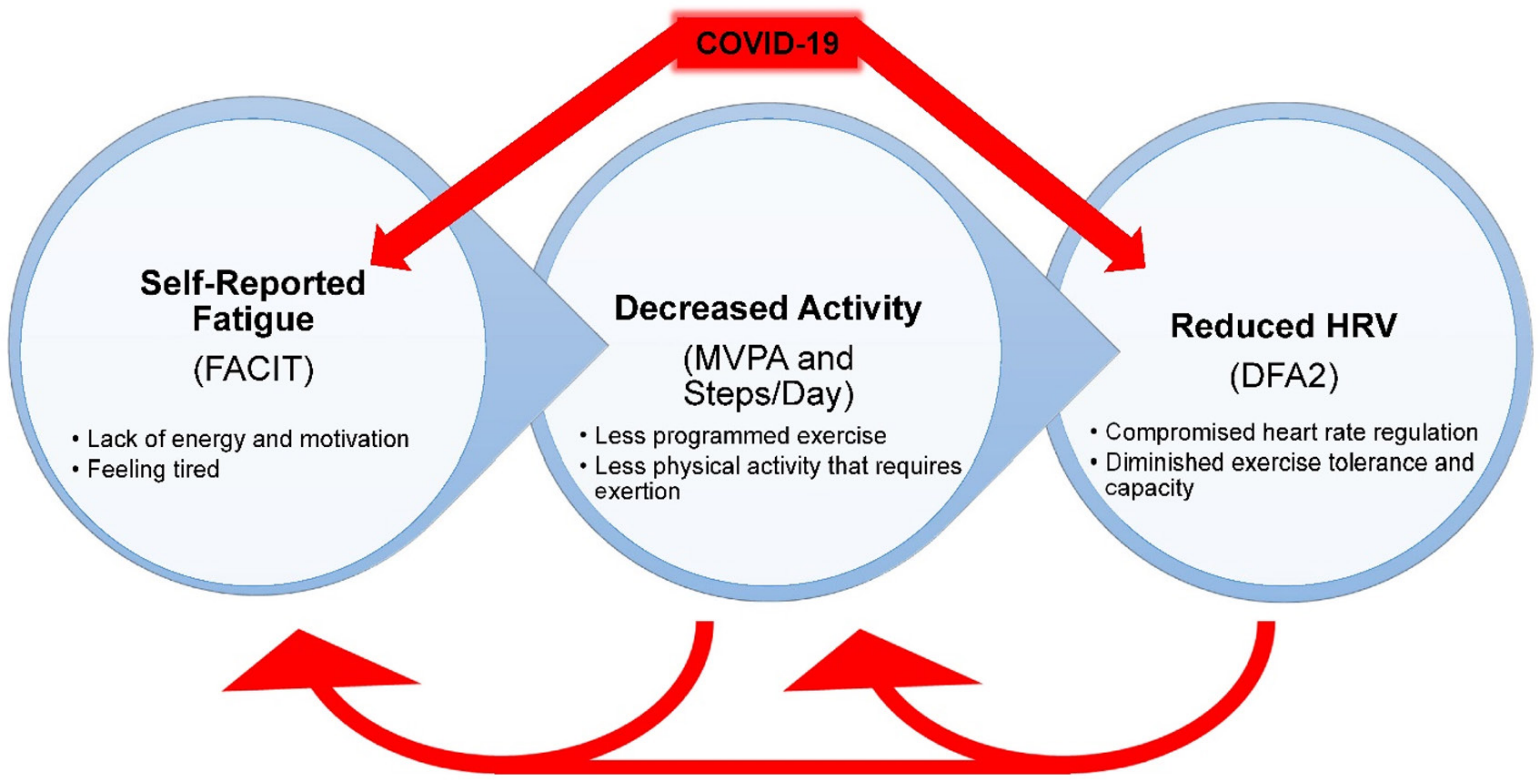
Theoretical model of fatigue, physical activity, and HRV in people with Long COVID. Linked variables with significant findings in the current study are shown in parentheses. COVID‐19 is associated with self‐reported fatigue and reductions in HRV. Fatigue is directly linked to physical activity and indirectly associated with HRV through the physical activity mediators. Reduced HRV could potentially lead to greater fatigue and/or decreased physical activity over time, secondary to exercise intolerance. Reduced physical activity may also increase perceptions of fatigue. The interrelationships of these variables and accompanying feedback loops encourage a continued decline in physical and ANS function in COVID‐19 survivors.

The combined results of this cross‐sectional study allowed us to generate a theoretical model of the proposed relation between self‐reported fatigue, PA, and HRV in COVID‐19 survivors (Figure 3). Building on other evidence of ANS dysfunction in COVID‐19 survivors, we showed that reduced HRV may be observed when compared to strictly matched controls. Furthermore, in this cohort of physically active survivors who did not all experience lingering symptoms, HRV was directly related to PA and indirectly connected to self‐reported fatigue through PA mediators. Our findings have overarching implications when considering that exercise and PA is a vital component of recovery from COVID‐19 and maintenance of long‐term health. We advise that survivors be treated cautiously as a special population with respect to exercise due to potential autonomic dysfunction, and that public health strategies designed to increase PA in this group should be sensitive to considerations of fatigue.

## AUTHOR CONTRIBUTIONS

All authors conceived and designed the research, performed the experiments, interpreted results of the experiments, edited and revised the manuscript, and approved the final version of the manuscript. MHH, LEO, and LMM analyzed the data, prepared the figures, and drafted the manuscript.

## FUNDING INFORMATION

This study was partially funded by a grant to SKH from the Institute for Women's Leadership at Marquette University.

## CONFLICT OF INTEREST STATEMENT

The authors of this study do not have any conflicts of interest to report.

## ETHICS STATEMENT

The study was approved by the institutional review board from Marquette University (Milwaukee, WI) and was conducted according to the Helsinki Declaration. All participants provided written informed consent.

## Supporting information




Table S1.
Table S2.
Table S3.

## Data Availability

The data that support the findings of this study are available from the corresponding author upon reasonable request.

## References

[phy215912-bib-0001] A clinical case definition of post COVID‐19 condition by a Delphi consensus . 2021. 10.1016/S1473-3099(21)00703-9 PMC8691845 34951953

[phy215912-bib-0002] Al‐Aly, Z. , Xie, Y. , & Bowe, B. (2021). High‐dimensional characterization of post‐acute sequelae of COVID‐19. Nature, 594(7862), 259–264. 33887749 10.1038/s41586-021-03553-9

[phy215912-bib-0003] Alkodaymi, M. S. , Omrani, O. A. , Fawzy, N. A. , Shaar, B. A. , Almamlouk, R. , Riaz, M. , Obeidat, M. , Obeidat, Y. , Gerberi, D. , Taha, R. M. , Kashour, Z. , Kashour, T. , Berbari, E. F. , Alkattan, K. , & Tleyjeh, I. M. (2022). Prevalence of post‐acute COVID‐19 syndrome symptoms at different follow‐up periods: A systematic review and meta‐analysis. Clinical Microbiology and Infection, 28(5), 657–666. 35124265 10.1016/j.cmi.2022.01.014 PMC8812092

[phy215912-bib-0004] Aparisi, A. , Ladrón, R. , Ybarra‐Falcón, C. , Tobar, J. , & San Román, J. A. (2022). Exercise intolerance in post‐acute sequelae of COVID‐19 and the value of cardiopulmonary exercise testing‐ a mini‐review. Frontiers in Medicine, 9, 924819. 35935782 10.3389/fmed.2022.924819 PMC9352932

[phy215912-bib-0005] Aragon‐Benedi, C. , et al. (2021). Is the heart rate variability monitoring using the analgesia nociception index a predictor of illness severity and mortality in critically ill patients with COVID‐19? A pilot study. PLoS ONE, 16(3), e0249128. 33760875 10.1371/journal.pone.0249128 PMC7990300

[phy215912-bib-0006] Asarcikli, L. D. , Hayiroglu, M. İ. , Osken, A. , Keskin, K. , Kolak, Z. , & Aksu, T. (2022). Heart rate variability and cardiac autonomic functions in post‐COVID period. Journal of Interventional Cardiac Electrophysiology, 63(3), 715–721. 35106678 10.1007/s10840-022-01138-8 PMC8806134

[phy215912-bib-0007] Barizien, N. , le Guen, M. , Russel, S. , Touche, P. , Huang, F. , & Vallée, A. (2021). Clinical characterization of dysautonomia in long COVID‐19 patients. Scientific Reports, 11(1), 14042. 34234251 10.1038/s41598-021-93546-5 PMC8263555

[phy215912-bib-0008] Bauer, A. , Kantelhardt, J. W. , Barthel, P. , Schneider, R. , Mäkikallio, T. , Ulm, K. , Hnatkova, K. , Schömig, A. , Huikuri, H. , Bunde, A. , Malik, M. , & Schmidt, G. (2006). Deceleration capacity of heart rate as a predictor of mortality after myocardial infarction: Cohort study. Lancet, 367(9523), 1674–1681. 16714188 10.1016/S0140-6736(06)68735-7

[phy215912-bib-0009] Berntson, G. G. , et al. (1997). Heart rate variability: Origins, methods, and interpretive caveats. Psychophysiology, 34(6), 623–648. 9401419 10.1111/j.1469-8986.1997.tb02140.x

[phy215912-bib-0010] Chen, L. Y. , et al. (2019). Cardiorespiratory fitness, adiposity, and heart rate variability: The coronary artery risk development in Young adults study. Medicine and Science in Sports and Exercise, 51(3), 509–514. 30277902 10.1249/MSS.0000000000001796 PMC6377325

[phy215912-bib-0011] (1996). Heart rate variability: Standards of measurement, physiological interpretation and clinical use. Task force of the European Society of Cardiology and the North American Society of Pacing and Electrophysiology. Circulation, 93(5), 1043–1065. 8598068

[phy215912-bib-0012] Collado‐Mateo, D. , Lavín‐Pérez, A. M. , Peñacoba, C. , Del Coso, J. , Leyton‐Román, M. , Luque‐Casado, A. , Gasque, P. , Fernández‐del‐Olmo, M. Á. , & Amado‐Alonso, D. (2021). Key factors associated with adherence to physical exercise in patients with chronic diseases and older adults: An umbrella review. International Journal of Environmental Research and Public Health, 18(4). 10.3390/ijerph18042023 PMC7922504 33669679

[phy215912-bib-0013] Craig, C. L. , et al. (2003). International physical activity questionnaire: 12‐country reliability and validity. Medicine and Science in Sports and Exercise, 35(8), 1381–1395. 12900694 10.1249/01.MSS.0000078924.61453.FB

[phy215912-bib-0014] Da Silveira, M. P. , Da Silva Fagundes, K. K. , Bizuti, M. R. , Starck, É. , Rossi, R. C. , & De Resende e Silva, D. T. (2021). Physical exercise as a tool to help the immune system against COVID‐19: An integrative review of the current literature. Clinical and Experimental Medicine, 21(1), 15–28. 32728975 10.1007/s10238-020-00650-3 PMC7387807

[phy215912-bib-0015] Dani, M. , Dirksen, A. , Taraborrelli, P. , Torocastro, M. , Panagopoulos, D. , Sutton, R. , & Lim, P. B. (2021). Autonomic dysfunction in'long COVID': Rationale, physiology and management strategies. Clinical Medicine (London, England), 21(1), e63–e67. 33243837 10.7861/clinmed.2020-0896 PMC7850225

[phy215912-bib-0016] Egerton, T. , Chastin, S. F. M. , Stensvold, D. , & Helbostad, J. L. (2016). Fatigue May contribute to reduced physical activity among older people: An observational study. The Journals of Gerontology. Series A, Biological Sciences and Medical Sciences, 71(5), 670–676. 26347508 10.1093/gerona/glv150

[phy215912-bib-0017] Galeoto, G. , Sansoni, J. , Valenti, D. , Mollica, R. , Valente, D. , Parente, M. , & Servadio, A. (2018). The effect of physiotherapy on fatigue and physical functioning in chronic fatigue syndrome patients: A systematic review. La Clinica Terapeutica, 169(4), e184–e188. 30151552 10.7417/T.2018.2076

[phy215912-bib-0018] Grissom, R. J. , & Kim, J. J. (2005). Effect Sizes For Research: A Broad Practical Approach. Lawrence Erlbaum Associates.

[phy215912-bib-0019] Guiraud, T. , et al. (2013). High‐intensity interval exercise improves vagal tone and decreases arrhythmias in chronic heart failure. Medicine and Science in Sports and Exercise, 45(10), 1861–1867. 23591293 10.1249/MSS.0b013e3182967559

[phy215912-bib-0020] Hasty, F. , García, G. , Dávila, H. , Wittels, S. H. , Hendricks, S. , & Chong, S. (2021). Heart rate variability as a possible predictive marker for acute inflammatory response in COVID‐19 patients. Military Medicine, 186(1–2), e34–e38. 33206183 10.1093/milmed/usaa405 PMC7717314

[phy215912-bib-0021] Hedges, L. V. , & Olkin, I. (1985). Statistical methods for meta‐analysis. Elsevier Science.

[phy215912-bib-0022] Henje Blom, E. , Olsson, E. M. G. , Serlachius, E. , Ericson, M. , & Ingvar, M. (2009). Heart rate variability is related to self‐reported physical activity in a healthy adolescent population. European Journal of Applied Physiology, 106(6), 877–883. 19479275 10.1007/s00421-009-1089-3 PMC2718191

[phy215912-bib-0023] Hilfiker, R. , Meichtry, A. , Eicher, M. , Nilsson Balfe, L. , Knols, R. H. , Verra, M. L. , & Taeymans, J. (2018). Exercise and other non‐pharmaceutical interventions for cancer‐related fatigue in patients during or after cancer treatment: A systematic review incorporating an indirect‐comparisons meta‐analysis. British Journal of Sports Medicine, 52(10), 651–658. 28501804 10.1136/bjsports-2016-096422 PMC5931245

[phy215912-bib-0024] Jurca, R. , Church, T. S. , Morss, G. M. , Jordan, A. N. , & Earnest, C. P. (2004). Eight weeks of moderate‐intensity exercise training increases heart rate variability in sedentary postmenopausal women. American Heart Journal, 147(5), e21. 15131556 10.1016/j.ahj.2003.10.024

[phy215912-bib-0025] Kaliyaperumal, D. , Rk, K. , Alagesan, M. , & Ramalingam, S. (2021). Characterization of cardiac autonomic function in COVID‐19 using heart rate variability: A hospital based preliminary observational study. Journal of Basic and Clinical Physiology and Pharmacology, 32(3), 247–253. 33705614 10.1515/jbcpp-2020-0378

[phy215912-bib-0026] Kleiger, R. E. , Miller, J. P. , Bigger, J. T., Jr. , & Moss, A. J. (1987). Decreased heart rate variability and its association with increased mortality after acute myocardial infarction. The American Journal of Cardiology, 59(4), 256–262. 3812275 10.1016/0002-9149(87)90795-8

[phy215912-bib-0027] Kluttig, A. , Schumann, B. , Swenne, C. A. , Kors, J. A. , Kuss, O. , Schmidt, H. , Werdan, K. , Haerting, J. , & Greiser, K. H. (2010). Association of health behaviour with heart rate variability: A population‐based study. BMC Cardiovascular Disorders, 10, 58. 21108803 10.1186/1471-2261-10-58 PMC3004825

[phy215912-bib-0028] Koenig, J. , & Thayer, J. F. (2016). Sex differences in healthy human heart rate variability: A meta‐analysis. Neuroscience and Biobehavioral Reviews, 64, 288–310. 26964804 10.1016/j.neubiorev.2016.03.007

[phy215912-bib-0029] Lakens, D. (2013). Calculating and reporting effect sizes to facilitate cumulative science: A practical primer for t‐tests and ANOVAs. Frontiers in Psychology, 4. 10.3389/fpsyg.2013.00863 PMC3840331 24324449

[phy215912-bib-0030] Lipponen, J. A. , & Tarvainen, M. P. (2019). A robust algorithm for heart rate variability time series artefact correction using novel beat classification. Journal of Medical Engineering & Technology, 43(3), 173–181. 31314618 10.1080/03091902.2019.1640306

[phy215912-bib-0031] Liu, X. , Xiang, L. , & Tong, G. (2020). Predictive values of heart rate variability, deceleration and acceleration capacity of heart rate in post‐infarction patients with LVEF >/=35. Annals of Noninvasive Electrocardiology, 25(6), e12771. 32633866 10.1111/anec.12771 PMC7679834

[phy215912-bib-0032] Luctkar‐Flude, M. F. , Groll, D. L. , Tranmer, J. E. , & Woodend, K. (2007). Fatigue and physical activity in older adults with cancer: A systematic review of the literature. Cancer Nursing, 30(5), E35–E45. 17876176 10.1097/01.NCC.0000290815.99323.75

[phy215912-bib-0033] Malik, M. (1996). Heart rate variability. Annals of Noninvasive Electrocardiology, 1(2), 151–181.

[phy215912-bib-0034] Manser, P. , Thalmann, M. , Adcock, M. , Knols, R. H. , & De Bruin, E. D. (2021). Can reactivity of heart rate variability Be a potential biomarker and monitoring tool to promote healthy aging? A systematic review with meta‐analyses. Frontiers in Physiology, 12, 686129. 34393813 10.3389/fphys.2021.686129 PMC8359814

[phy215912-bib-0035] McGraw, K. O. , & Wong, S. P. (1992). A common language effect size statistic. Psychological Bulletin, 111, 361–365.

[phy215912-bib-0036] Meinhardt, J. , Radke, J. , Dittmayer, C. , Franz, J. , Thomas, C. , Mothes, R. , Laue, M. , Schneider, J. , Brünink, S. , Greuel, S. , Lehmann, M. , Hassan, O. , Aschman, T. , Schumann, E. , Chua, R. L. , Conrad, C. , Eils, R. , Stenzel, W. , Windgassen, M. , … Heppner, F. L. (2021). Olfactory transmucosal SARS‐CoV‐2 invasion as a port of central nervous system entry in individuals with COVID‐19. Nature Neuroscience, 24(2), 168–175. 33257876 10.1038/s41593-020-00758-5

[phy215912-bib-0037] Mol, M. B. A. , Strous, M. T. A. , Van Osch, F. H. M. , Vogelaar, F. J. , Barten, D. G. , Farchi, M. , Foudraine, N. A. , & Gidron, Y. (2021). Heart‐rate‐variability (HRV), predicts outcomes in COVID‐19. PLoS ONE, 16(10), e0258841. 34710127 10.1371/journal.pone.0258841 PMC8553073

[phy215912-bib-0038] Nalbandian, A. , Sehgal, K. , Gupta, A. , Madhavan, M. V. , McGroder, C. , Stevens, J. S. , Cook, J. R. , Nordvig, A. S. , Shalev, D. , Sehrawat, T. S. , Ahluwalia, N. , Bikdeli, B. , Dietz, D. , Der‐Nigoghossian, C. , Liyanage‐Don, N. , Rosner, G. F. , Bernstein, E. J. , Mohan, S. , Beckley, A. A. , … Wan, E. Y. (2021). Post‐acute COVID‐19 syndrome. Nature Medicine, 27(4), 601–615. 10.1038/s41591-021-01283-z PMC8893149 33753937

[phy215912-bib-0039] (2020). Long COVID: Let patients help define long‐lasting COVID symptoms. Nature, 586(7828), 170. 33029005 10.1038/d41586-020-02796-2

[phy215912-bib-0040] Pan, Y. , Yu, Z. , Yuan, Y. , Han, J. , Wang, Z. , Chen, H. , Wang, S. , Wang, Z. , Hu, H. , Zhou, L. , Lai, Y. , Zhou, Z. , Wang, Y. , Meng, G. , Yu, L. , & Jiang, H. (2021). Alteration of autonomic nervous system is associated with severity and outcomes in patients with COVID‐19. Frontiers in Physiology, 12, 630038. 34093217 10.3389/fphys.2021.630038 PMC8170133

[phy215912-bib-0041] Pavlov, V. A. , & Tracey, K. J. (2017). Neural regulation of immunity: Molecular mechanisms and clinical translation. Nature Neuroscience, 20(2), 156–166. 28092663 10.1038/nn.4477

[phy215912-bib-0042] Phillips, S. , & Williams, M. A. (2021). Confronting our next National Health Disaster ‐ long‐haul Covid. The New England Journal of Medicine, 385(7), 577–579. 34192429 10.1056/NEJMp2109285

[phy215912-bib-0043] Puetz, T. W. (2006). Physical activity and feelings of energy and fatigue: Epidemiological evidence. Sports Medicine, 36(9), 767–780. 16937952 10.2165/00007256-200636090-00004

[phy215912-bib-0044] Puetz, T. W. , Flowers, S. S. , & O'Connor, P. J. (2008). A randomized controlled trial of the effect of aerobic exercise training on feelings of energy and fatigue in sedentary young adults with persistent fatigue. Psychotherapy and Psychosomatics, 77(3), 167–174. 18277063 10.1159/000116610

[phy215912-bib-0045] Raman, B. , Bluemke, D. A. , Lüscher, T. F. , & Neubauer, S. (2022). Long COVID: Post‐acute sequelae of COVID‐19 with a cardiovascular focus. European Heart Journal, 43(11), 1157–1172. 35176758 10.1093/eurheartj/ehac031 PMC8903393

[phy215912-bib-0046] Ramirez‐Velez, R. , et al. (2020). Effect of moderate‐ versus high‐intensity interval exercise training on heart rate variability parameters in inactive Latin‐American adults: A randomized clinical trial. Journal of Strength and Conditioning Research, 34(12), 3403–3415. 28198783 10.1519/JSC.0000000000001833

[phy215912-bib-0047] Rzepka, M. , Toś, M. , Boroń, M. , Gibas, K. , & Krzystanek, E. (2020). Relationship between fatigue and physical activity in a polish cohort of multiple sclerosis patients. Medicina (Kaunas, Lithuania), 56(12), 726. 33371510 10.3390/medicina56120726 PMC7767485

[phy215912-bib-0048] Saffer, H. , Dave, D. , Grossman, M. , & Ann Leung, L. (2013). Racial, ethnic, and gender differences in physical activity. Journal of Human Capital, 7(4), 378–410. 25632311 10.1086/671200 PMC4306423

[phy215912-bib-0049] Sallis, R. , Young, D. R. , Tartof, S. Y. , Sallis, J. F. , Sall, J. , Li, Q. , Smith, G. N. , & Cohen, D. A. (2021). Physical inactivity is associated with a higher risk for severe COVID‐19 outcomes: A study in 48 440 adult patients. British Journal of Sports Medicine, 55(19), 1099–1105. 33849909 10.1136/bjsports-2021-104080

[phy215912-bib-0050] Sandercock, G. R. , Bromley, P. D. , & Brodie, D. A. (2005). Effects of exercise on heart rate variability: Inferences from meta‐analysis. Medicine and Science in Sports and Exercise, 37(3), 433–439. 15741842 10.1249/01.mss.0000155388.39002.9d

[phy215912-bib-0051] Shaffer, F. , & Ginsberg, J. P. (2017). An overview of heart rate variability metrics and norms. Frontiers in Public Health, 5, 258. 29034226 10.3389/fpubh.2017.00258 PMC5624990

[phy215912-bib-0052] Shaffer, F. , McCraty, R. , & Zerr, C. L. (2014). A healthy heart is not a metronome: An integrative review of the heart's anatomy and heart rate variability. Frontiers in Psychology, 5, 1040. 25324790 10.3389/fpsyg.2014.01040 PMC4179748

[phy215912-bib-0053] Sjoberg, N. , Brinkworth, G. D. , Wycherley, T. P. , Noakes, M. , & Saint, D. A. (1985). Moderate weight loss improves heart rate variability in overweight and obese adults with type 2 diabetes. Journal of Applied Physiology, 110(4), 1060–1064. 10.1152/japplphysiol.01329.2010 21212252

[phy215912-bib-0054] Solinski, M. , et al. (2022). Heart rate variability comparison between young males after 4‐6 weeks from the end of SARS‐CoV‐2 infection and controls. Scientific Reports, 12(1), 8832. 35614330 10.1038/s41598-022-12844-8 PMC9130989

[phy215912-bib-0055] Stute, N. L. , Stickford, J. L. , Province, V. M. , Augenreich, M. A. , Ratchford, S. M. , & Stickford, A. S. L. (2021). COVID‐19 is getting on our nerves: Sympathetic neural activity and haemodynamics in young adults recovering from SARS‐CoV‐2. The Journal of Physiology, 599(18), 4269–4285. 34174086 10.1113/JP281888 PMC8447023

[phy215912-bib-0056] Tarvainen, M. P. , Niskanen, J. P. , Lipponen, J. A. , Ranta‐aho, P. O. , & Karjalainen, P. A. (2014). Kubios HRV—Heart rate variability analysis software. Computer Methods and Programs in Biomedicine, 113(1), 210–220. 24054542 10.1016/j.cmpb.2013.07.024

[phy215912-bib-0057] Thaweethai, T. , Jolley, S. E. , Karlson, E. W. , Levitan, E. B. , Levy, B. , McComsey, G. A. , McCorkell, L. , Nadkarni, G. N. , Parthasarathy, S. , Singh, U. , Walker, T. A. , Selvaggi, C. A. , Shinnick, D. J. , Schulte, C. C. M. , Atchley‐Challenner, R. , Horwitz, L. I. , Foulkes, A. S. , RECOVER Consortium Authors , RECOVER Consortium , … Zisis, S. (2023). Development of a definition of Postacute sequelae of SARS‐CoV‐2 infection. JAMA, 329(22), 1934–1946. 37278994 10.1001/jama.2023.8823 PMC10214179

[phy215912-bib-0058] Thayer, J. F. , Yamamoto, S. S. , & Brosschot, J. F. (2010). The relationship of autonomic imbalance, heart rate variability and cardiovascular disease risk factors. International Journal of Cardiology, 141(2), 122–131. 19910061 10.1016/j.ijcard.2009.09.543

[phy215912-bib-0059] Wagoner, C. W. , Lee, J. T. , & Battaglini, C. L. (2021). Community‐based exercise programs and cancer‐related fatigue: A systematic review and meta‐analysis. Support Care Cancer, 29(9), 4921–4929. 33751225 10.1007/s00520-021-06135-7

[phy215912-bib-0060] Xie, Y. , Xu, E. , Bowe, B. , & al‐Aly, Z. (2022). Long‐term cardiovascular outcomes of COVID‐19. Nature Medicine, 28(3), 583–590. 10.1038/s41591-022-01689-3 PMC8938267 35132265

[phy215912-bib-0061] Yellen, S. B. , Cella, D. F. , Webster, K. , Blendowski, C. , & Kaplan, E. (1997). Measuring fatigue and other anemia‐related symptoms with the functional assessment of cancer therapy (FACT) measurement system. Journal of Pain and Symptom Management, 13(2), 63–74. 9095563 10.1016/s0885-3924(96)00274-6

[phy215912-bib-0062] Yin, C. , Li, J. , Wang, Z. , Zhi, Y. , & Xu, L. (2023). Decreased heart rate variability in COVID‐19. Intensive Care Research, 3(1), 87–91. 36471860 10.1007/s44231-022-00024-1 PMC9713139

[phy215912-bib-0063] Zanin, A. , Amah, G. , Chakroun, S. , Testard, P. , Faucher, A. , le, T. Y. V. , Slama, D. , le Baut, V. , Lozeron, P. , Salmon, D. , & Kubis, N. (2023). Parasympathetic autonomic dysfunction is more often evidenced than sympathetic autonomic dysfunction in fluctuating and polymorphic symptoms of "long‐COVID" patients. Scientific Reports, 13(1), 8251. 37217645 10.1038/s41598-023-35086-8 PMC10201474

