# Supplementary material for: Heart rate variability is reduced in COVID‐19 survivors and associated with physical activity and fatigue
Source: Physiol Rep. 2024 Jan 19;12(2):e15912. doi: 10.14814/phy2.15912 (PMC10799199; doi:10.14814/phy2.15912)
Supplement: Supplementary file 1 — Table S1. Table S2. Table S3. [file PHY2-12-e15912-s001.docx]

**SUPPLEMENTAL TABLES**

| **Supplemental Table S1. COVID-19 Symptom Checklist** |
| --- |
| *Cough (new onset or worsening of chronic cough)*  *Shortness of breath*  *Fever (temperature > 100 degrees Fahrenheit*  *Chills*  *Sore throat*  *Runny nose*  *Muscle pain*  *Headache*  *Loss of taste*  *Loss of smell*  *COVID toes (swelling and discoloration of the toes)*  *Chest pain*  *Body rash*  *Hair loss*  *Anemia*  *Joint pain*  *Brain fog (difficulty with thinking, concentration, memory)*  *Fatigue*  *Depression*  *Anxiety*  *Changes in mood*  *Heart palpitations (fast-beating or pounding heart)*  *Other** |
| *Open-ended question to allow free response |

| **Supplemental Table S2. HRV Comparison between COVID-19 Survivors and Controls** | | | | | | | |
| --- | --- | --- | --- | --- | --- | --- | --- |
| ***Analysis Domain*** | ***Variable**** | | ***COV (n=41)*** | ***CON (n=41)*** | ***Sig.***  ***(P)*** | ***Hedges’ g*** | ***CL (%)*** |
| ***Time*** | *Mean RR Interval (ms)* | | 973 ± 212 | 991 ± 154 | 0.444 | 0.10 | 52.7 |
|  | *SDNN (ms)* | | 44.8 ± 26.4 | 58.9 ± 38.5 | 0.082 | 0.42 | 61.9 |
|  | *RMSSD (ms)* | | 48.9 ± 36.8 | 59.2 ± 46.4 | 0.206 | 0.24 | 56.9 |
|  | *NN50 (beats)* | | 73.8 ± 81.0 | 82.0 ± 68.0 | 0.328 | 0.11 | 53.1 |
|  | *pNN50 (%)* | | 23.8 ± 23.4 | 28.0 ± 23.1 | 0.297 | 0.18 | 55.1 |
|  | *Triangular Index* | | 10.0 ± 4.3 | 12.4 ± 6.3 | 0.096 | 0.44 | 62.3 |
|  | *TINN (ms)* | | 226.7 ± 120.8 | 286.7 ± 170.7 | 0.089 | 0.40 | 61.2 |
|  | *AC (ms)* | | -19.6 ± 13.8 | -26.4 ± 16.4 | 0.041 | 0.45 | 62.4 |
|  | *DC (ms)* | | 20.7 ± 17.1 | 30.8 ± 24.0 | 0.032 | 0.48 | 63.4 |
| ***Frequency*** | *Peak Frequency (Hz)* | *HF* | 0.233 ± 0.059 | 0.202 ± 0.049 | 0.019 | 0.57 | 65.7 |
|  |  | *LF* | 0.089 ± 0.033 | 0.096 ± 0.028 | 0.292 | 0.23 | 56.4 |
|  |  | *VLF* | 0.036 ± 0.005 | 0.036 ± 0.005 | 0.580 | 0.11 | 53.1 |
|  | *Absolute Power (ms^2^)* | *HF* | 1306 ± 2162 | 1756 ± 2762 | 0.297 | 0.18 | 55.1 |
|  |  | *LF* | 1071 ± 1471 | 2462 ± 3517 | 0.042 | 0.51 | 64.2 |
|  |  | *VLF* | 84.2 ± 79.7 | 79.3 ± 67.2 | 0.893 | 0.07 | 51.9 |
|  | *Relative Power (%)* | *HF* | 44.8 ± 22.3 | 40.1 ± 22.7 | 0.100 | 0.21 | 55.9 |
|  |  | *LF* | 48.5 ± 20.3 | 56.0 ± 22.3 | 0.288 | 0.35 | 60.0 |
|  |  | *VLF* | 6.65 ± 5.78 | 3.86 ± 3.51 | 0.012 | 0.58 | 66.0 |
|  | *Normalized Power* | *HF* | 47.5 ± 22.3 | 41.7 ± 23.1 | 0.216 | 0.25 | 57.2 |
|  |  | *LF* | 52.5 ± 22.3 | 58.3 ± 23.1 | 0.216 | 0.25 | 57.2 |
|  | *Total Power (ms^2^)* | | 2462 ± 3427 | 4298 ± 5374 | 0.071 | 0.40 | 61.3 |
|  | *LF/HF* | | 2.75 ± 6.41 | 2.95 ± 4.14 | 0.216 | 0.04 | 51.0 |
| ***Non-Linear*** | *SD1 (ms)* | | 34.6 ± 26.0 | 41.9 ± 32.9 | 0.206 | 0.24 | 56.9 |
|  | *SD2 (ms)* | | 52.0 ± 28.8 | 70.9 ± 44.9 | 0.047 | 0.50 | 63.8 |
|  | *SD2/SD1* | | 1.80 ± 0.68 | 1.89 ± 0.58 | 0.328 | 0.14 | 54.0 |
|  | *Approximate Entropy* | | 1.062 ± 0.087 | 1.036 ± 0.122 | 0.337 | 0.24 | 56.9 |
|  | *Sample Entropy* | | 1.646 ± 0.239 | 1.573 ± 0.345 | 0.361 | 0.24 | 56.9 |
|  | *Correlation Dimension* | | 1.980 ± 1.625 | 2.242 ± 1.513 | 0.663 | 0.17 | 54.7 |
|  | *DFA1* | | 0.967 ± 0.334 | 1.025 ±0.290 | 0.356 | 0.18 | 55.2 |
|  | *DFA2* | | 0.297 ± 0.144 | 0.214 ± 0.090 | 0.004 | 0.68 | 68.8 |
| ms: milliseconds; Hz: hertz | | | | | |  |  |

| **Supplemental Table S3. Significance (*P*) of Fatigue, Physical Activity, and Symptomology Predictors in HRV Regression Models from COVID-19 Survivors Controlling for Age, Sex, and Body Fat Percentage** | | | | | | | |
| --- | --- | --- | --- | --- | --- | --- | --- |
| ***Predictor*** | ***AC (ms)*** | ***DC (ms)*** | ***HF Peak (Hz)*** | ***LF Power (ms^2^)*** | ***VLF Power (%)*** | ***SD2 (ms)*** | ***DFA2*** |
| *FACIT* | 0.785 | 0.865 | 0.839 | 0.933 | 0.346 | 0.816 | 0.801 |
| *Sedentary Time (min/day)* | 0.316 | 0.169 | 0.737 | 0.227 | 0.235 | 0.454 | 0.729 |
| *Light Activity (min/day)* | 0.092 | 0.791 | 0.502 | 0.929 | 0.705 | 0.659 | 0.627 |
| *MVPA (min/day)* | 0.334 | 0.459 | 0.170 | 0.454 | 0.443 | 0.435 | **0.003** |
| *Steps Per Day* | 0.515 | 0.661 | 0.982 | 0.864 | 0.306 | 0.746 | **0.007** |
| *Total Acute Symptoms (N)* | 0.175 | 0.186 | 0.139 | 0.454 | 0.335 | 0.332 | 0.455 |
| *Acute Ranked Symptom Severity*  *(0-4)* | 0.226 | 0.231 | 0.156 | 0.542 | 0.418 | 0.452 | 0.567 |
| *Total Lingering Symptoms (N)* | 0.290 | 0.271 | 0.319 | 0.323 | 0.225 | 0.262 | 0.171 |
| *Lingering Ranked Symptom Severity*  *(0-4)* | 0.155 | 0.146 | 0.168 | 0.128 | 0.229 | 0.108 | 0.156 |
| *Pre-Infection Physical Activity (IPAQ)* | 0.569 | 0.744 | 0.527 | 0.608 | 0.866 | 0.708 | 0.965 |
| *Current Physical Activity (IPAQ)* | 0.126 | 0.231 | 0.270 | 0.781 | 0.231 | 0.407 | 0.892 |
| *Physical Activity Difference (Current – Pre-Infection)* | 0.752 | 0.709 | 0.906 | 0.597 | 0.628 | 0.972 | 0.896 |
| FACIT: Functional Assessment of Chronic Illness Therapy – Fatigue Scale  MVPA: moderate/vigorous physical activity  IPAQ: International Physical Activity Questionnaire | | | | | | | |
